# Immune-Mediated Inflammation May Contribute to the Pathogenesis of Cardiovascular Disease in Mucopolysaccharidosis Type I

**DOI:** 10.1371/journal.pone.0150850

**Published:** 2016-03-17

**Authors:** Omar Khalid, Moin U. Vera, Philip L. Gordts, N. Matthew Ellinwood, Philip H. Schwartz, Patricia I. Dickson, Jeffrey D. Esko, Raymond Y. Wang

**Affiliations:** 1 National Human Neural Stem Cell Resource, CHOC Research Institute, Orange, California, United States of America; 2 Division of Medical Genetics, Los Angeles Biomedical Research Institute at Harbor-UCLA, Torrance, California, United States of America; 3 University of California-San Diego, La Jolla, California, United States of America; 4 Iowa State University College of Agriculture and Life Sciences, Ames, Iowa, United States of America; 5 Sue and Bill Gross Stem Cell Research Center, University of California-Irvine, Irvine, California, United States of America; 6 Division of Metabolic Disorders, CHOC Children’s Hospital, Orange, California, United States of America; University of Pennsylvania, UNITED STATES

## Abstract

**Background:**

Cardiovascular disease, a progressive manifestation of α-L-iduronidase deficiency or mucopolysaccharidosis type I, continues in patients both untreated and treated with hematopoietic stem cell transplantation or intravenous enzyme replacement. Few studies have examined the effects of α-L-iduronidase deficiency and subsequent glycosaminoglycan storage upon arterial gene expression to understand the pathogenesis of cardiovascular disease.

**Methods:**

Gene expression in carotid artery, ascending, and descending aortas from four non-tolerized, non-enzyme treated 19 month-old mucopolysaccharidosis type I dogs was compared with expression in corresponding vascular segments from three normal, age-matched dogs. Data were analyzed using R and whole genome network correlation analysis, a bias-free method of categorizing expression level and significance into discrete modules. Genes were further categorized based on module-trait relationships. Expression of clusterin, a protein implicated in other etiologies of cardiovascular disease, was assessed in canine and murine mucopolysaccharidosis type I aortas via Western blot and *in situ* immunohistochemistry.

**Results:**

Gene families with more than two-fold, significant increased expression involved lysosomal function, proteasome function, and immune regulation. Significantly downregulated genes were related to cellular adhesion, cytoskeletal elements, and calcium regulation. Clusterin gene overexpression (9-fold) and protein overexpression (1.3 to 1.62-fold) was confirmed and located specifically in arterial plaques of mucopolysaccharidosis-affected dogs and mice.

**Conclusions:**

Overexpression of lysosomal and proteasomal-related genes are expected responses to cellular stress induced by lysosomal storage in mucopolysaccharidosis type I. Upregulation of immunity-related genes implicates the potential involvement of glycosaminoglycan-induced inflammation in the pathogenesis of mucopolysaccharidosis-related arterial disease, for which clusterin represents a potential biomarker.

## Introduction

Mucopolysaccharidosis type I (MPS I), caused by a deficiency of the lysosomal enzyme α-L-iduronidase (IDUA), results in shortened lifespan, multisystemic somatic involvement, and variable neurocognitive degeneration because of accumulation of heparan sulfate (HS) and dermatan sulfate (DS) glycosaminoglycan (GAG) substrates in body tissues such as brain, soft tissues, chondrocytes, liver, and spleen [1]. Cardiovascular disease is a cardinal manifestation of MPS I, characterized by progressive thickening and compromised function of the heart valves, left ventricular hypertrophy, and diffuse coronary artery stenosis [2–6].

The advent of treatments to replace the missing IDUA enzyme, whether with intravenous enzyme replacement therapy (ERT) or via hematopoietic stem cell transplant (HSCT), has enabled MPS I patients to survive into adulthood [7, 8]. Although ERT and HSCT are able to mitigate many symptoms of MPS I, clinical experience has demonstrated that these treatments attenuate, but do not cure, the disease. Certain tissues remain resistant to treatment and continue to manifest GAG storage. Consequently, as MPS I patients survive into adulthood they face a different set of potentially life-threatening disease complications such as those involving the cardiovascular system [9, 10]. Cardiac sudden death, left-sided valvular insufficiency, ventricular dysfunction, and coronary intimal proliferation with stenosis all have been reported in stably treated patients [11–14]. Accumulation of GAG within cardiovascular structures in the face of ongoing treatment is the likely origin of these symptoms, as well as childhood-onset carotid intima-media thickening and abnormally reduced elasticity [15–19].

The pathogenesis and etiologies of treatment resistance of cardiovascular disease in MPS I are not well characterized, but studies in the murine and canine models of the disease indicate that accumulation of undegraded HS and DS GAG in the heart, valves, and vasculature alone do not adequately describe the pathophysiology. Both untreated and treated MPS I canines develop similar cardiovascular findings to human MPS I, with cardiac hypertrophy, nodular valve thickening, and vascular smooth muscle proliferation of the aorta with luminal stenosis [20–22]. Detailed histopathology of canine aortic lesions demonstrates vascular smooth muscle proliferation, activated CD68^+^ macrophages, and fragmented elastin fibrils in addition to GAG storage [23]. The murine MPS I model also manifests with cardiac enlargement, valvular thickening and dysfunction, and dilatation of the aorta with vascular wall thickening with elastin fibril degradation [24, 25].

Gene expression studies are a useful method to identify potential mechanisms of disease progression, but have not been comprehensively assessed for cardiovascular disease in any model of MPS I. The primary focus of expression studies for the mucopolysaccharidoses has been neurodegeneration in the Sanfilippo syndromes (MPS III) and Sly syndrome (MPS VII) [26–30]. Assessment of aortic mRNA expression for dogs with MPS I and VII, and mice with MPS VII has centered on quantification of cytokine, complement, and other inflammation-related genes [31–34]. Herein, we report alterations in arterial gene and protein expression in the canine MPS I model system, the identification of a potential marker for MPS I cardiovascular disease, and data supporting the hypothesis that a GAG-induced inflammatory process is responsible for the pathogenesis of MPS I cardiovascular disease.

## Materials and Methods

### Test animals and husbandry

This study was reviewed and approved by the Institutional Animal Care and Use Committees of both Iowa State University (IACUC #12-04-5791-K) and the Los Angeles Biomedical Research Institute at Harbor-UCLA (LA BioMed IACUC #20013–01). Studies were conducted in compliance with both UDSA and NIH guidelines for the use of dogs in research. Four MPS I (IDUA^-/-^) canines were produced from artificial insemination breedings of parental stock, diagnosed via α-L-iduronidase enzyme assay and PCR, and maintained at Iowa State University until 1 year of age, after which they were transported to LA BioMed, an AAALAC accredited facility overseen by a licensed veterinarian. Animals had *ad libitum* access to food (standard Teklad canine lab chow) and water, housing with enrichment, and a 12 hour light/dark cycle. Housing consisted of expanded mesh flooring, and involved housing with compatible conspecifics as required for social species unless medically or behaviorally contraindicated. Animal care was provided by the Laboratory Animal Resources veterinary staff of the respective institutions. The dog colony has a null mutation in intron 1 of the canine α-L-iduronidase gene that results in abnormal mRNA splicing, introduces a premature termination codon, and completely eliminates IDUA protein expression [35]. The four dogs were neither tolerized nor treated with IV rhIDUA, and had received six monthly intra-articular rhIDUA injections as previously reported [36].

Mouse-related protocols were approved by the University of California San Diego IACUC Animal Subjects Committee (IACUC #S99127). The MPS I (*Idua*^-/-^) mouse colony was housed, maintained, and veterinarian-supervised at The University of California San Diego, an AAALAC accredited facility. The colony, which is on the C57BL/6 background, was originally obtained from the Jackson Laboratories (B6.129-Idua^tm1Clk/J^) and was bred locally for 9 generations. *Idua* genotyping was performed as per S1 Appendix. Mice utilized for this study were fed standard chow and were neither tolerized nor treated with any form of rhIDUA.

### Tissue collection and euthanasia

At 18 months of age, dogs were euthanized with a 1 cc / 10 lb dose of euthasol and 1 cm rings from the ascending aorta, descending aorta proximal to the renal arteries, and common carotid arteries were immediately obtained post-mortem. Arterial rings were processed as follows: 1) one section from each site was embedded in a cryomold with OCT (Tissue-Tek CRYO-OCT Compound, ThermoFisher Scientific, Waltham, MA), frozen and stored at -80°C, 2) one section placed in TRIzol solution (Life Technologies, Grand Island, NY) for subsequent RNA extraction, 3) and one section frozen and stored at -80°C for subsequent Western blotting.

At 9–12 months of age, female MPS I (mean age, 9.4 months; n = 8) and age-matched wild-type (mean age, 10.2 months n = 3) mice were euthanized with a combination of Isoflurane inhalation (Abbott, North Chicago, IL), cervical dislocation, and exsanguination. Heart and aorta collection was performed as per Daugherty and Whitman, and summarized as follows: after exsanguination, the right atrium was incised and the animal was perfused with 20 mL cold phosphate buffered saline (PBS) through the left ventricle [37]. The abdominal viscera were removed and the heart and aorta were carefully dissected *en bloc* to the iliac bifurcation. The heart was cut from the ascending aorta approximately 5 mm from the point of emergence from the left ventricle, embedded in a cryomold with OCT, and stored frozen at -80°C.

### RNA isolation, amplification, and labeling

RNA isolation from canine aortic specimens was performed using the TRIzol RNA Purification System (Life Technologies, Grand Island, NY). RNA concentration and quality, as well as dye incorporation efficiency, was checked with an ND-1000 spectrophotometer (NanoDrop Technologies, Rockland, DE, USA) and the Agilent Bioanalyzer 2100. A total of 100 ng RNA was used as initial starting template, which was labeled with Cy-3 or Cy-5 cytidine 5’-triphosphate using the low input fluorescent linear amplification kit (Agilent, Santa Clara, CA).

### Microarray hybridizations and data analysis

Comparisons of canine arterial gene expression between control and untreated MPS animals were conducted with a canine-specific microarray covering 43,803 probes (Agilent G2519F 4x44k, Santa Clara, CA), for a total of four comparison groups: MPS ascending aorta vs. control ascending aorta, MPS descending aorta vs. control descending aorta, MPS carotid artery vs. control carotid artery, and finally pooled MPS artery (ascending aorta, descending aorta, carotid artery) vs. pooled control artery. Each comparison used four pairs of MPS vs corresponding age- and gender- matched animals to produce four biologic replicates.

### Bioinformatic analysis

The data was analyzed by the WGCNA package in R 3.0.2, a package we have used previously to analyze large datasets and identify novel genomic targets [38–41]. Co-expression networks were formed on genes with similar behavior. Trait information (e.g. ascending aorta, carotid artery, and descending aorta) and Pearson correlation data were obtained.

Database for Annotation, Visualization and Integrated Discovery (DAVID) v6.7 was used to functionally annotate the genes that clustered together [42,43]. DAVID’s functional annotation tool allows for pathway analysis using the Kyoto Encyclopedia of Genes and Genomes (KEGG), gene ontology annotation for biological processes, molecular function, and cellular components, assessment of transcription factor binding sites, and identification of tissues matching the gene clusters. All relationships determined had corresponding statistics within DAVID. The functional annotations presented here had a p-value ≤ 0.05 with Bonferroni correction.

Network analysis was performed using two methods. The first was WGCNA to identify networks without any prior knowledge. This enabled discovery of novel nodes and edges that could be visualized using Cytoscape version 3.1.1 [44]. The second was with GeneMania (a plugin available in Cytoscape) to examine connections based on prior evidence including co-expression, shared protein domains, pathway, physical interactions, genetic interactions, and co-localization [39, 45].

### Western blotting

Flash frozen sections of harvested ascending and descending aorta that had been harvested from homozygous MPS I and heterozygous carrier dogs and stored at -80°C were thawed on ice. Sections of approximately 30 to 50 mg of each tissue sample were cut and mixed in a 1:3 weight to volume ratio with lysis buffer containing 4% SDS, EDTA, a protease inhibitor cocktail (P8340, Sigma-Aldrich, St. Louis, MO) and phosphatase inhibitor cocktails (P0044 and P5726, Sigma-Aldrich, St. Louis, MO). Standard Dounce homogenization was performed while each tissue sample was kept on ice in a 4°C room. After homogenization, each sample was sonicated in a 4°C water bath for 1 hour and then centrifuged at 10,000 rpm for 10 minutes. The supernatant was collected and centrifuged again at 10,000 rpm for 10 minutes. This supernatant was collected and total protein content of each sample was determined with a standard colorimetric assay (Bio-Rad, Philadelphia, PA) converting absorbance measured as optical density units at 595 nm (Shimadzu BioSpec 1601 Double Beam UV-Visible Spectrophotometer, Shimadzu Scientific Instruments, Columbia, MD) to protein concentration using bovine serum albumin as a standard.

Samples were diluted to a final concentration of 4 μg/μL by addition of the appropriate volume of a standard Laemmli loading buffer. These were then heated to 95°C for 20 minutes prior to loading onto a gel (Novex Tris-Glycine 4–20% Mini Protein Gel, Life Technologies, Grand Island, NY). Each well was loaded with 20 μg of total protein and a protein ladder covering the size range from 10 to 180 kDa (PageRuler Prestained Protein Ladder, Life Technologies, Grand Island, NY) was loaded in one well of each gel for reference. Separation was performed via 4–20% Tris-Glycine SDS-PAGE followed by transfer to a nitrocellulose membrane (BioTrace NT nitrocellulose transfer membrane, Pall Corp., Port Washington, NY).

Standard immunoblotting was then performed with mouse monoclonal anti-human clusterin alpha chain (1:1,000 or 1 ng/μL; EMD Millipore, Billerica, MA) diluted in 2.5% nonfat milk and incubated overnight at 4°C. After washing, the membrane was incubated for one hour at room temperature with goat anti-mouse IgG HRP conjugated secondary antibody (1:50,000; Southern Biotechnology, Birmingham, AL, USA) diluted in 2.5% nonfat milk, and then detected with Immobilon Chemiluminescent HRP Substrate (EMD Millipore, Billerica, MA). Membranes were then stripped, washed and re-probed with mouse monoclonal anti-human smooth muscle alpha-actin (1:10,000; Dako North America, Inc., Carpinteria, CA) diluted in 2.5% nonfat milk. After washing, the membrane was incubated for one hour at room temperature with goat anti-mouse IgG HRP conjugated secondary antibody (1:100,000; Southern Biotechnology, Birmingham, AL) diluted in 2.5% nonfat milk, and then detected with Immobilon Chemiluminescent HRP Substrate (EMD Millipore, Billerica, MA).

Western blot band intensities were quantified using ImageJ. After conversion of Western blot images to black and white and subtraction of background, the signal intensities of clusterin bands were subsequently measured and normalized to signal intensities of corresponding α-actin bands. The ratio of mean normalized MPS I clusterin intensity to mean heterozygote clusterin intensity was calculated to determine fold-change expression in ascending and descending aorta homogenates.

### Canine aorta immunohistochemistry

Transverse cuts through each OCT-embedded tissue sample were taken with a cryostat at -20°C to produce 12 micron-thick cross-sectional slices that were mounted on glass slides. Slide mounted tissue sections were covered with neutral PBS at room temperature for twenty minutes, rinsed, and then fixed with 4% paraformaldehyde in PBS for 20 minutes. Slides were washed in PBS and then each tissue section was blocked with hydrogen peroxide for 20 minutes. After washing in PBS each tissue section was then blocked with 5% normal goat serum in PBS for one hour at room temperature. Tissue sections were then incubated overnight at 4°C with mouse monoclonal anti-human clusterin alpha chain antibody (1:2,500 or 0.4 ng/μl; EMD Millipore, Billerica, MA) diluted in 3% BSA in neutral PBS, mouse monoclonal anti-human smooth muscle alpha actin antibody (1:2,500; Dako North America, Inc., Carpinteria, CA) diluted in 3% BSA in neutral PBS, or 3% BSA in neutral PBS alone. Detection of specific staining was performed with an anti-mouse HRP-conjugated secondary antibody and diaminobenzidine (DAB) substrate-chromogen (EXPOSE Mouse and Rabbit HRP/DAB Detection Kit, Abcam, Cambridge, MA) according to manufacturer’s protocol. Tissue sections were counterstained with hematoxylin prior to visualization. Cover slips were applied to each slide and standard bright field optical microscopy was performed. In the transverse aorta sections positive (DAB) signal appears brown while the counterstain appears blue.

### Murine aorta preparation and immunohistochemistry

6 micron-thick cross-sections of heart and aorta were obtained from OCT-embedded samples with a cryostat at -20°C, mounted on glass slides, dried at room temperature for 30 minutes and then fixed in fresh 10% neutral buffered formalin (Fisher #SF94-4, Waltham, MA) for 15 minutes. Slides were washed 3 times in PBS with Tween-20® (PBST). Endogenous peroxidases were blocked with a 0.03% solution of hydrogen peroxide in PBST, then washed another 3 times in PBST. Slides were overlaid with 1% BSA in PBST (Sigma #A4503, St Louis, MO) for 10 minutes, endogenous biotin blocked with Vector Labs #SP-2001 kit, and washed another 3 times in PBST. Slides were then overlaid with 1% BSA in PBST and polyclonal goat anti-mouse clusterin alpha chain (1:100; Santa Cruz #SC-6420, Dallas, TX), incubated at room temperature for 1 hour, and washed 3 times in PBST. Secondary staining was accomplished with biotinylated donkey anti-goat antibody (1:500; Jackson ImmunoResearch Laboratories #805-065-180, West Grove, PA) incubated 30 minutes at room temperature, then washed 3 times in PBST. Slides were then incubated with streptavidin-HRP (1:500; Jackson ImmunoResearch Laboratories #016-030-084, West Grove, PA) for 30 minutes, rinsed, then developed with 3-amino-9-ethylcarbazole chromogen (Vector Labs #SK-4200, Burlingame, CA) for 10 minutes. Slides were counterstained in Mayer’s Hematoxylin (Sigma #MHS16, St Louis, MO) for 1 minute, washed in PBST, air dried, and cover slips applied with aqueous mounting media (Vector Labs #H-5501, Burlingame, CA).

## Results

**Fig 1:**
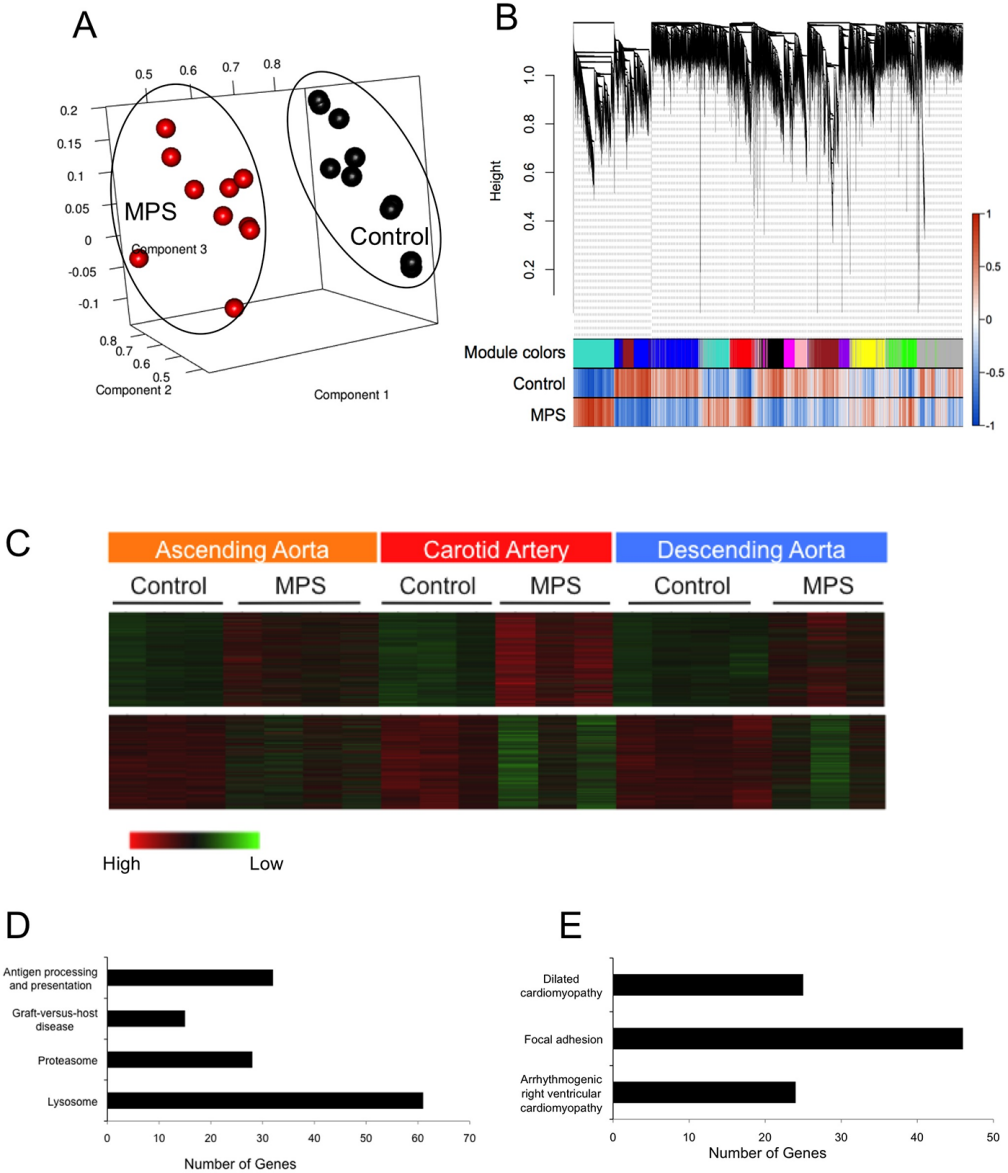
Microarray Results From IDUA^-/-^ Canine Arterial Samples. A. Principal component analysis of IDUA^-/-^ canine arteries compared to control canine arteries clearly shows the formation of two sample clusters separating IDUA^-/-^ and control expression. B. WGCNA dendrogram on the microarray samples showing different modules related to the Pearson correlation of the IDUA^-/-^ and control samples. C. Heatmaps of the two major modules (turquoise and blue) formed by WGCNA showing genes upregulated (top panel) or downregulated (bottom panel) by IDUA deficiency. D. Genes significantly upregulated in IDUA^-/-^ canine arteries include those related to immunity / inflammation (KEGG pathways: antigen processing and presentation, graft-versus-host disease), proteasomal function, and lysosomal function. E. Genes significantly downregulated in IDUA^-/-^ canine arteries are primarily related to cellular adhesion and the cytoskeleton (placed into the dilated cardiomyopathy, focal adhesion, arrhythmogenic right ventricular cardiomyopathy KEGG pathways).

Microarray was performed on IDUA^-/-^ and control (IDUA^+/-^) canine arterial samples obtained from common carotid artery, ascending aorta, and descending aorta. Data was initially analyzed using principal component analysis (PCA). Two clusters were visualized, clearly differentiating the IDUA^-/-^ arterial expression patterns from the control patterns (Fig 1A). Dendrograms and trait heatmaps were also made using the adjacency matrix technique (S1 Fig). Using both of these dimension reduction methods, it can easily be determined if the samples cluster together or if there are outliers. Again, two main clusters were observed, one for IDUA^-/-^ and the other for control. In addition, expression patterns from different animals at the same arterial sites demonstrated clustering according to IDUA genotype. In other words, carotid artery expression in the MPS I animals clustered together and was distinct from both MPS I ascending aorta expression and descending aorta expression patterns.

Data was subsequently analyzed using whole genome correlation network analysis (WGCNA) to identify specific modules that related back to the MPS I trait. Use of this method loosens the stringency in the analysis, since groups of genes are examined together allows a significance matrix to be made based on the networks formed [46]. Genes were further categorized based on fold-change and additional statistical tests within each module. The lists of probes/genes 2-fold or more upregulated or downregulated compared to control across modules can be found in Tables 1 and 2, respectively; full listings are included in S1 and S2 Tables. Subsequently, a dendrogram was made to represent the modules formed with the Pearson correlations for each trait shown (Fig 1B). There were ten total modules that formed (S2 Fig). Specific attention was paid to the turquoise and blue modules because those were the most statistically significant modules representing genes consistently upregulated in all three sampled vascular segments of the IDUA^-/-^ dogs (upper panel, Fig 1C), or genes consistently downregulated in all three sampled vascular segments of the IDUA^-/-^ dogs (lower panel, Fig 1C). Further downstream analysis with a volcano plot to identify the top targets within the turquoise and blue modules is seen in S3 Fig.

**Table 1:** Upregulated Genes in IDUA^-/-^ Canine Arteries. The 50 upregulated genes with significant (p-value ≤ 0.05) and greater than 2-fold level of upregulation in IDUA^-/-^ canine arteries, compared to controls. Note that some genes are represented more than once due to existence of multiple mRNA probes per gene. For the full list of significantly upregulated genes, please refer to S1 Table.

Fold Change	Gene Symbol	Systematic Name	Description
27.126	GPNMB	XM_858105	PREDICTED: Canis familiaris similar to glycoprotein (transmembrane) nmb isoform b precursor, transcript variant 3 (LOC482355), mRNA [XM_858105]
20.245	GPNMB	XM_858105	PREDICTED: Canis familiaris similar to glycoprotein (transmembrane) nmb isoform b precursor, transcript variant 3 (LOC482355), mRNA [XM_858105]
12.432	ENSCAFT00000039657	ENSCAFT00000039657	Osteopontin (Fragment)
11.926	CSTB	XM_535601	PREDICTED: Canis familiaris similar to Cystatin B
11.566	ENSCAFT00000039661	ENSCAFT00000039661	Osteopontin (Fragment)
10.506	DLA-DRA1	ENSCAFT00000001254	MHC class II DR alpha chain
10.220	CLU	NM_001003370	Canis lupus familiaris clusterin (CLU)
9.595	CLU	NM_001003370	Canis lupus familiaris clusterin (CLU)
9.464	CTSK	ENSCAFT00000035613	Cathepsin K precursor
8.985	TC71707	TC71707	Q49RE5_CANFA (Q49RE5) Cytochrome b, partial (35%)
7.766	CSTB	XM_535601	PREDICTED: Canis familiaris similar to Cystatin B
7.587	LOC479746	XM_536874	PREDICTED: Canis familiaris similar to Ferritin light chain 2
7.022	TC57368	TC57368	HUMLPLSTN2 L-plastin {Homo sapiens}
6.975	GPNMB	XM_858105	PREDICTED: Canis familiaris similar to glycoprotein (transmembrane) nmb isoform b precursor, transcript variant 3 (LOC482355), mRNA [XM_858105]
6.968	CLU	NM_001003370	Canis lupus familiaris clusterin (CLU)
6.947	CD86	NM_001003146	Canis lupus familiaris CD86 molecule (CD86)
6.769	DR104862	DR104862	Canine cardiovascular system biased cDNA
6.738	FTL	NM_001024636	Canis lupus familiaris ferritin, light polypeptide
6.718	CTSS	NM_001002938	Canis lupus familiaris cathepsin S (CTSS)
6.704	CLU	NM_001003370	Canis lupus familiaris clusterin (CLU)
6.677	APOE	XM_847504	PREDICTED: Canis familiaris apolipoprotein E, transcript variant 2 (APOE)
6.666	CTSK	NM_001033996	Canis lupus familiaris cathepsin K (CTSK)
6.655	CTSK	NM_001033996	Canis lupus familiaris cathepsin K (CTSK)
6.605	LGMN	XM_537355	PREDICTED: Canis familiaris similar to Legumain precursor
6.453	CTSK	NM_001033996	Canis lupus familiaris cathepsin K (CTSK)
6.423	DLA-DRB1	NM_001014768	Canis lupus familiaris MHC class II DLA DRB1 beta chain (DLA-DRB1)
6.193	FTL	NM_001024636	Canis lupus familiaris ferritin, light polypeptide (FTL)
6.178	CTSK	NM_001033996	Canis lupus familiaris cathepsin K (CTSK)
6.055	TYRP1	XM_859450	PREDICTED: Canis familiaris tyrosinase-related protein 1 (TYRP1)
6.026	CTSK	NM_001033996	Canis lupus familiaris cathepsin K (CTSK)
6.013	BPI	XM_534417	PREDICTED: Canis familiaris similar to Bactericidal permeability-increasing protein precursor (BPI)
6.006	CTSB	XM_543203	PREDICTED: Canis familiaris similar to cathepsin B preproprotein
5.833	TC50899	TC50899	Q9N2I5_FELCA (Q9N2I5) CD16
5.828	TC62836	TC62836	FCU92795 fusin {Felis catus}
5.727	LGMN	XM_537355	PREDICTED: Canis familiaris similar to Legumain precursor
5.680	DLA-DQB1	NM_001014381	Canis lupus familiaris MHC class II, DQ beta 1 (DLA-DQB1)
5.660	CSGALNACT1	XM_539946	PREDICTED: Canis familiaris similar to chondroitin beta1,4 N-acetylgalactosaminyltransferase
5.651	MT2A	NM_001003149	Canis lupus familiaris metallothionein 2A (MT2A)
5.493	TC51307	TC51307	Unknown
5.331	TGM2	XM_542991	PREDICTED: Canis familiaris similar to Protein-glutamine gamma-glutamyltransferase
5.306	SMOC2	XM_858080	PREDICTED: Canis familiaris similar to secreted modular calcium-binding protein 2
5.190	ASAH1	XM_540012	PREDICTED: Canis familiaris similar to N-acylsphingosine amidohydrolase (acid ceramidase)
5.152	APOE	ENSCAFT00000007432	Apolipoprotein E (Apo-E)
5.104	TC54889	TC54889	Unknown
5.069	DLA-DRA1	NM_001011723	Canis lupus familiaris MHC class II DR alpha chain (DLA-DRA1)
4.984	RRAGD	XM_532231	PREDICTED: Canis familiaris similar to Ras-related GTP binding D
4.921	E02824	E02824	Canis familiaris cDNA clone LIB30321_005_G04
4.912	RRAGD	XM_532231	PREDICTED: Canis familiaris similar to Ras-related GTP binding D
4.902	TYROBP	XM_533687	PREDICTED: Canis familiaris similar to TYRO protein tyrosine kinase binding protein isoform 2 precursor
4.882	FTH1	ENSCAFT00000025200	Ferritin heavy chain (EC 1.16.3.1) (Ferritin H subunit)

**Table 2:** Down-regulated Genes in IDUA^-/-^ Canine Arteries. The 50 down-regulated genes with significant (p-value ≤ 0.05) and greater than 2-fold level of down-regulation in IDUA^-/-^ canine arteries, compared to controls. Again, some genes are represented more than once due to existence of multiple mRNA probes per gene. For the full list of significantly down-regulated genes, please refer to S2 Table.

Fold Change	Gene Symbol	Systematic Name	Description
4.948	TC67209	TC67209	HUMRPS17A S17 ribosomal protein {Homo sapiens}
3.970	LRRK2	XM_543734	PREDICTED: Canis familiaris similar to leucine-rich repeat kinase 2
3.666	CNN1	XM_862565	PREDICTED: Canis familiaris similar to calponin 1, basic, smooth muscle, transcript variant 4
3.392	CO611926	CO611926	DG9-109j22 DG9-ovary Canis familiaris cDNA 3'
3.309	SMTN	XM_860837	PREDICTED: Canis familiaris similar to smoothelin isoform c
3.218	NRGN	XM_536537	PREDICTED: Canis familiaris similar to neurogranin (LOC479401)
3.212	CSRP1	XM_843516	PREDICTED: Canis familiaris similar to Cysteine and glycine-rich protein 1
3.157	TCAP	XM_858636	PREDICTED: Canis familiaris similar to Telethonin (Titin cap protein)
3.109	PPP1R12B	XM_843384	PREDICTED: Canis familiaris similar to Protein phosphatase 1 regulatory subunit 12B (Myosin phosphatase targeting subunit 2)
3.018	DN756599	DN756599	GL-Cf-13395 GLGC-LIB0001-cf Canis familiaris Normalized Mixed Tissue cDNA
2.949	LOC488984	XM_843847	PREDICTED: Canis familiaris similar to Actin, alpha skeletal muscle
2.865	AB012223	AB012223	Canis familiaris LINE-1 element ORF2 mRNA
2.855	ENSCAFT00000034839	ENSCAFT00000034839	NADH-ubiquinone oxidoreductase chain 3
2.836	PPP1R12B	XM_843384	PREDICTED: Canis familiaris similar to Protein phosphatase 1 regulatory subunit 12B (Myosin phosphatase targeting subunit 2)
2.812	ENSCAFT00000034824	ENSCAFT00000034824	NADH-ubiquinone oxidoreductase chain 2 (EC 1.6.5.3) (NADH dehydrogenase subunit 2)
2.812	BM538907	BM538907	hb02c06.g1 Canis cDNA from testes cells Canis familiaris cDNA clone
2.782	LOC488984	XM_843847	PREDICTED: Canis familiaris similar to Actin, alpha skeletal muscle
2.764	TC55776	TC55776	Unknown
2.761	TAGLN	XM_856022	PREDICTED: Canis familiaris similar to transgelin, transcript variant 3
2.734	FHL1	XM_549282	PREDICTED: Canis familiaris similar to four and a half LIM domains 1
2.712	LOC488984	XM_843847	PREDICTED: Canis familiaris similar to Actin, alpha skeletal muscle
2.705	OGN	XM_848247	PREDICTED: Canis familiaris similar to Mimecan precursor (Osteoglycin)
2.692	DMD	NM_001003343	Canis lupus familiaris dystrophin (muscular dystrophy, Duchenne and Becker types)
2.673	TC68286	TC68286	Unknown
2.630	ENSCAFT00000022802	ENSCAFT00000022802	Fibronectin (FN) (Fragment)
2.626	SLC22A3	XM_533467	PREDICTED: Canis familiaris similar to solute carrier family 22 member 3
2.617	LOC488984	XM_843847	PREDICTED: Canis familiaris similar to Actin, alpha skeletal muscle
2.598	DN879822	DN879822	nae29h08.y1 Dog eye eye minus lens and cornea. Unnormalized (nae)
2.594	PTGS1	NM_001003023	Canis lupus familiaris prostaglandin-endoperoxide synthase 1
2.586	LOC491592	XM_548713	PREDICTED: Canis familiaris similar to ankyrin repeat domain 26
2.567	PPAP2B	XM_536696	PREDICTED: Canis familiaris similar to phosphatidic acid phosphatase type 2B
2.526	PPAP2B	XM_536696	PREDICTED: Canis familiaris similar to phosphatidic acid phosphatase type 2B
2.489	C1QTNF1	XM_843609	PREDICTED: Canis familiaris similar to C1q and tumor necrosis factor related protein 1
2.481	TPM1	ENSCAFT00000026876	Tropomyosin (Fragment)
2.460	MYH11	XM_857511	PREDICTED: Canis familiaris similar to smooth muscle myosin heavy chain 11 isoform SM1
2.459	ADCY5	M88649	Canis familiaris adenylyl cyclase type V
2.458	LOC480803	XM_537918	PREDICTED: Canis familiaris similar to ankyrin repeat domain 26
2.429	TC48376	TC48376	MALAT_HUMAN (Q9UHZ2) Metastasis-associated lung adenocarcinoma transcript 1
2.407	CF412534	CF412534	Canine heart normalized cDNA Library, pBluescript Canis familiaris cDNA clone CH3#080_C09 5'
2.405	DN368051	DN368051	LIB3731-002-Q6-K7-H5 LIB3731 Canis familiaris cDNA clone CLN2470649
2.393	CN005552	CN005552	ip32c07.g1 Brain—Cerebellum Library (DOGEST8) Canis familiaris cDNA clone ip32c07
2.363	CO586466	CO586466	DG2-130p9 DG2-brain Canis familiaris
2.346	ENSCAFT00000022799	ENSCAFT00000022799	Fibronectin (FN) (Fragment)
2.331	TCAP	XM_858636	PREDICTED: Canis familiaris similar to Telethonin (Titin cap protein)
2.322	LOC488991	XM_843985	PREDICTED: Canis familiaris similar to Protein FAM13C1
2.309	PDZRN4	XM_543731	PREDICTED: Canis familiaris similar to PDZ domain containing RING finger protein 4 (Ligand of Numb-protein X 4)
2.301	LOC607572	XM_844308	PREDICTED: Canis familiaris similar to ankyrin repeat domain 26
2.286	PPP1R12A	XM_859971	PREDICTED: Canis familiaris similar to Protein phosphatase 1 regulatory subunit 12A (Myosin phosphatase targeting subunit 1)
2.286	TC73854	TC73854	Unknown
2.280	A_11_P0000024197	Unknown	TC47203

The pathways significantly implicated by the upregulated gene module (turquoise; Fig 1 D) include antigen processing and presentation, graft-versus-host disease, proteasome, and the lysosome. Specific genes from each upregulated pathway are detailed in Table 3. The pathways significantly implicated by the downregulated gene module (blue; Fig 1 E) include dilated cardiomyopathy, focal adhesion, and arrhythmogenic right ventricular cardiomyopathy and are detailed in Table 4. These identified pathways are consistent with pathways identified from prior expression arrays from MPS IIIb mouse brain [26, 30], MPS VII mouse brain [29] and liver [47].

**Table 3:** Upregulated Canine Arterial IDUA^-/-^ Genes Sorted by Category. Upregulated arterial genes in IDUA^-/-^ canines fall into categories associated with lysosomal function, proteasomal function, and the immune system (graft-versus-host disease and antigen processing).

Ontology Category		
*"LYSOSOMAL FUNCTION"*		
Gene Symbol	Gene Name	Entrez Gene ID
ATP6V0C	ATPase, H+ transporting, lysosomal 16kDa, V0 subunit c	479877
ATP6V0B	ATPase, H+ transporting, lysosomal 21kDa, V0 subunit b	482528
ATP6V0D1	ATPase, H+ transporting, lysosomal 38kDa, V0 subunit d1	479685
ATP6V1H	ATPase, H+ transporting, lysosomal 50/57kDa, V1 subunit H	486953
ATP6V0A1	ATPase, H+ transporting, lysosomal V0 subunit a1	607705
ATP6AP1	ATPase, H+ transporting, lysosomal accessory protein 1	610922
CD63	CD63 molecule	474391
GM2A	GM2 ganglioside activator	479324
NAGA	N-acetylgalactosaminidase, alpha	481226
GNPTAB	N-acetylglucosamine-1-phosphate transferase, alpha and beta subunits	475443
NAGPA	N-acetylglucosamine-1-phosphodiester alpha-N-acetylglucosaminidase	490019
NAGLU	N-acetylglucosaminidase, alpha-	490965
ASAH1	N-acylsphingosine amidohydrolase (acid ceramidase) 1	482897
SGSH	N-sulfoglucosamine sulfohydrolase	403707
NPC1	Niemann-Pick disease, type C1	403698
TCIRG1	T-cell, immune regulator 1, ATPase, H+ transporting, lysosomal V0 subunit A3	483691
AP3B2	adaptor-related protein complex 3, beta 2 subunit	479053
AP3M1	adaptor-related protein complex 3, mu 1 subunit	489052
AP3S2	adaptor-related protein complex 3, sigma 2 subunit	479042
AP4B1	adaptor-related protein complex 4, beta 1 subunit	483125
ARSA	arylsulfatase A	474457
AGA	aspartylglucosaminidase	475638
CTSA	cathepsin A	611146
CTSB	cathepsin B	486077
CTSC	cathepsin C	403458; 478608
CTSD	cathepsin D	483662
CTSG	cathepsin G	608543
CTSH	cathepsin H	479065
CTSK	cathepsin K	608843
CTSL2	cathepsin L2	403708
CTSS	cathepsin S	403400
CTSZ	cathepsin Z	611983
CLTA	clathrin, light chain (Lca)	474765
CTNS	cystinosis, nephropathic	491220
DNASE2	deoxyribonuclease II, lysosomal	476697
GLB1	galactosidase, beta 1	403873
GBA	glucosidase, beta; acid (includes glucosylceramidase)	612206
GUSB	glucuronidase, beta	403831
GGA2	golgi associated, gamma adaptin ear containing, ARF binding protein 2	608555
HGSNAT	heparan-alpha-glucosaminide N-acetyltransferase	482833
HEXA	hexosaminidase A (alpha polypeptide)	487633
HEXB	hexosaminidase B (beta polypeptide)	478100
LGMN	legumain	480232
LIPA	lipase A, lysosomal acid, cholesterol esterase	610650
LAPTM5	lysosomal multispanning membrane protein 5	487324
LAPTM4A	lysosomal protein transmembrane 4 alpha	475678
LAMP1	lysosomal-associated membrane protein 1	476995
LAMP2	lysosomal-associated membrane protein 2	481037
M6PR	mannose-6-phosphate receptor (cation dependent)	477700
MAN2B1	mannosidase, alpha, class 2B, member 1; similar to UPF0139 protein CGI-140	476703; 484932
MANBA	mannosidase, beta A, lysosomal	487883
PPT1	palmitoyl-protein thioesterase 1	475316
PPT2	palmitoyl-protein thioesterase 2	474856
PSAP	prosaposin	479240
SCARB2	scavenger receptor class B, member 2	478435
NEU1	sialidase 1 (lysosomal sialidase)	481717
AP1S2	similar to Adapter-related protein complex 1 sigma 1B subunit (Sigma-adaptin 1B)	611468
SLC11A1	solute carrier family 11 (proton-coupled divalent metal ion transporters), member 1	478909
SLC17A5	solute carrier family 17 (anion/sugar transporter), member 5	474969
SORT1	sortilin 1	479915
TPP1	tripeptidyl peptidase I	485337
*"PROTEASOMAL FUNCTION"*		
Gene Symbol	Gene Name	Entrez Gene ID
PSMC1	proteasome (prosome, macropain) 26S subunit, ATPase, 1	478703
PSMC2	proteasome (prosome, macropain) 26S subunit, ATPase, 2	475896
PSMC3	proteasome (prosome, macropain) 26S subunit, ATPase, 3	475980
PSMC5	proteasome (prosome, macropain) 26S subunit, ATPase, 5	480478
PSMD11	proteasome (prosome, macropain) 26S subunit, non-ATPase, 11	480610
PSMD14	proteasome (prosome, macropain) 26S subunit, non-ATPase, 14	478765
PSMD2	proteasome (prosome, macropain) 26S subunit, non-ATPase, 2	478654
PSMD3	proteasome (prosome, macropain) 26S subunit, non-ATPase, 3	491018
PSMD6	proteasome (prosome, macropain) 26S subunit, non-ATPase, 6	484700
PSMD8	proteasome (prosome, macropain) 26S subunit, non-ATPase, 8	476470
PSME1	proteasome (prosome, macropain) activator subunit 1 (PA28 alpha)	480256
PSME2	proteasome (prosome, macropain) activator subunit 2 (PA28 beta)	480258
PSME4	proteasome (prosome, macropain) activator subunit 4	474594
PSMA4	proteasome (prosome, macropain) subunit, alpha type, 4	475132
PSMA5	proteasome (prosome, macropain) subunit, alpha type, 5	490123
PSMA6	proteasome (prosome, macropain) subunit, alpha type, 6	480290
PSMA7	proteasome (prosome, macropain) subunit, alpha type, 7	404305
PSMB1	proteasome (prosome, macropain) subunit, beta type, 1	475040
PSMB10	proteasome (prosome, macropain) subunit, beta type, 10	489749
PSMB2	proteasome (prosome, macropain) subunit, beta type, 2	475338
PSMB3	proteasome (prosome, macropain) subunit, beta type, 3	474987; 480537
PSMB4	proteasome (prosome, macropain) subunit, beta type, 4	475848
PSMB5	proteasome (prosome, macropain) subunit, beta type, 5	480246
PSMB8	proteasome (prosome, macropain) subunit, beta type, 8	474865
PSMB9	proteasome (prosome, macropain) subunit, beta type, 9	474867
POMP	proteasome maturation protein	477325
PSMA1	similar to Proteasome subunit alpha type 1 (Proteasome component C2)	476867; 608388; 610188; 612667; 613000
PSMA3	similar to Proteasome subunit alpha type 3 (Proteasome component C8)	480338; 607261
*"GRAFT-VS-HOST"*		
Gene Symbol	Gene Name	Entrez Gene ID
CD80	CD80 molecule	403765
CD86	CD86 molecule	403764
DLA-12 / DLA-64	MHC class I DLA-12; MHC class I DLA-64	474838; 541592
DLA88	MHC class I DLA-88	474836
DLA-DRB1	MHC class II DLA DRB1 beta chain	474860
DLA-DRA1	MHC class II DR alpha chain	481731
DLA-79	MHC class Ib	483594
IL1A	interleukin 1, alpha	403782
IL1B	interleukin 1, beta	403974
IL2	interleukin 2	403989
IL6	interleukin 6 (interferon, beta 2)	403985
HLA-DMA	major histocompatibility complex, class II, DM alpha	481732
HLA-DOB	major histocompatibility complex, class II, DO beta	607786
DLA-DQA1	major histocompatibility complex, class II, DQ alpha 1	474861
DLA-DQB1	major histocompatibility complex, class II, DQ beta 1	474862
*"ANTIGEN PROCESSING"*		
Gene Symbol	Gene Name	Entrez Gene ID
DLA-12 / DLA-64	MHC class I DLA-12; MHC class I DLA-64	474838; 541592
DLA88	MHC class I DLA-88	474836
DLA-DRB1	MHC class II DLA DRB1 beta chain	474860
DLA-DRA1	MHC class II DR alpha chain	481731
DLA-79	MHC class Ib	483594
TAPBP	TAP binding protein (tapasin)	481740
B2M	beta-2-microglobulin	478284
CANX	calnexin	403908
CTSB	cathepsin B	486077
CTSS	cathepsin S	403400
HSPA1L	heat shock 70kDa protein 1-like	474850
HSP70	heat shock protein 70	403612
HSPA4	heat shock protein Apg-2	474680
KLRD1	killer cell lectin-like receptor subfamily D, member 1	611360
LGMN	legumain	480232
HLA-DMA	major histocompatibility complex, class II, DM alpha	481732
HLA-DOB	major histocompatibility complex, class II, DO beta	607786
DLA-DQA1	major histocompatibility complex, class II, DQ alpha 1	474861
DLA-DQB1	major histocompatibility complex, class II, DQ beta 1	474862
NFYB	nuclear transcription factor Y, beta	475450
NFYC	nuclear transcription factor Y, gamma	475312
PSME1	proteasome (prosome, macropain) activator subunit 1 (PA28 alpha)	480256
PSME2	proteasome (prosome, macropain) activator subunit 2 (PA28 beta)	480258
RFXANK	regulatory factor X-associated ankyrin-containing protein	476662
LOC480726	similar to 78 kDa glucose-regulated protein precursor (Endoplasmic reticulum lumenal Ca(2+) binding protein grp78)	480726
LOC476669	similar to Gamma-interferon inducible lysosomal thiol reductase precursor (Gamma-interferon-inducible protein IP-30)	476669
LOC479329	similar to HLA class II histocompatibility antigen, gamma chain (HLA-DR antigens associated invariant chain) (Ia antigen-associated invariant chain)	479329
LOC607470	similar to Heat shock protein HSP 90-alpha (HSP 86)	607470
LOC490008	similar to MHC class II transactivator	490008
HSPA8	similar to heat shock 70kDa protein 8 isoform 2; heat shock 70kDa protein 8; similar to Heat shock cognate 71 kDa protein (Heat shock 70 kDa protein 8); similar to heat shock protein 8	479406; 607182; 608802
LOC480438	similar to heat shock protein 1, alpha	480438
LOC608885	similar to heat shock protein 8	608885

**Table 4:** Down-regulated Canine Arterial IDUA^-/-^ Genes Sorted by Category. Down-regulated arterial genes in IDUA^-/-^ dogs fall into categories associated with cytoskeletal proteins, cellular adhesion, and ion channels (termed arrhythmogenic right ventricular cardiomyopathy, focal adhesion, and dilated cardiomyopathy).

Ontology Category		
*"ARRHYTHMOGENIC RIGHT VENTRICULAR CARDIOMYOPATHY"*		
Systematic Name	Gene Name	Entrez Gene ID
ACTB	actin, beta	610787
ACTN1	actinin, alpha 1	480369
ACTN2	actinin, alpha 2	479191
ACTN4	actinin, alpha 4	484526
LOC442937	beta-actin	442937
CACNA1S	calcium channel, voltage-dependent, L type, alpha 1S subunit	490244
CACNB1	calcium channel, voltage-dependent, beta 1 subunit	491030
CACNB2	calcium channel, voltage-dependent, beta 2 subunit	487113
CACNB4	calcium channel, voltage-dependent, beta 4 subunit	609361
CTNNA1	catenin (cadherin-associated protein), alpha 2	483088
CTNNA3	catenin (cadherin-associated protein), alpha 3	489008
DES	desmin	497091
DSC2	desmocollin 2	403860
DMD	dystrophin (muscular dystrophy, Duchenne and Becker types)	606758
GJA1	gap junction protein, alpha 1, 43kDa	403418
ITGA5	integrin, alpha 5 (fibronectin receptor, alpha polypeptide)	486493
ITGA6	integrin, alpha 6	478800
ITGA8	integrin, alpha 8	487119
ITGA9	integrin, alpha 9	477021
ITGB4	integrin, beta 4	483318
ITGB8	integrin, beta 8	475253
RYR2	ryanodine receptor 2 (cardiac)	403615
SGCG	sarcoglycan, gamma (35kDa dystrophin-associated glycoprotein)	486043
LOC492249	similar to Emerin	492249
*"DILATED CARDIOMYOPATHY"*		
Systematic Name	Gene Name	Entrez Gene ID
GNAS	GNAS complex locus	403943
ACTB	actin, beta	610787
ADCY2	adenylate cyclase 2 (brain)	478624
ADCY5	adenylate cyclase 5	403859
ADCY9	adenylate cyclase 9	490031
LOC442937	beta-actin	442937
CACNA1S	calcium channel, voltage-dependent, L type, alpha 1S subunit	490244
CACNB1	calcium channel, voltage-dependent, beta 1 subunit	491030
CACNB2	calcium channel, voltage-dependent, beta 2 subunit	487113
CACNB4	calcium channel, voltage-dependent, beta 4 subunit	609361
DES	desmin	497091
DMD	dystrophin (muscular dystrophy, Duchenne and Becker types)	606758
ITGA5	integrin, alpha 5 (fibronectin receptor, alpha polypeptide)	486493
ITGA6	integrin, alpha 6	478800
ITGA8	integrin, alpha 8	487119
ITGA9	integrin, alpha 9	477021
ITGB4	integrin, beta 4	483318
ITGB8	integrin, beta 8	475253
PLN	phospholamban	414755
RYR2	ryanodine receptor 2 (cardiac)	403615
SGCG	sarcoglycan, gamma (35kDa dystrophin-associated glycoprotein)	486043
LOC492249	similar to Emerin	492249
TTN	titin	478819
TPM3	tropomyosin 3; tropomyosin 1 (alpha); similar to tropomyosin 1 alpha chain isoform 4	478332; 480137; 484695; 491970
TNF	tumor necrosis factor (TNF superfamily, member 2)	403922
*"FOCAL ADHESION"*		
Systematic Name	Gene Name	Entrez Gene ID
RAP1A	RAP1A, member of RAS oncogene family	483225
RAPGEF1	Rap guanine nucleotide exchange factor (GEF) 1	491286
ARHGAP5	Rho GTPase activating protein 5	490642
ROCK1	Rho-associated, coiled-coil containing protein kinase 1	480181
ACTB	actin, beta	610787
ACTN1	actinin, alpha 1	480369
ACTN2	actinin, alpha 2	479191
ACTN4	actinin, alpha 4	484526
LOC442937	beta-actin	442937
CAV1	caveolin 1, caveolae protein, 22kDa	403980
CAV2	caveolin 2	403611; 475294
COL1A1	collagen, type I, alpha 1	403651
COL1A2	collagen, type I, alpha 2	403824
COL2A1	collagen, type II, alpha 1	403826
COL4A1	collagen, type IV, alpha 1	403496
COL5A1	collagen, type V, alpha 1	480684
COL6A3	collagen, type VI, alpha 3	403582
FLNC	filamin C, gamma (actin binding protein 280)	482266
ITGA5	integrin, alpha 5 (fibronectin receptor, alpha polypeptide)	486493
ITGA6	integrin, alpha 6	478800
ITGA8	integrin, alpha 8	487119
ITGA9	integrin, alpha 9	477021
ITGB4	integrin, beta 4	483318
ITGB8	integrin, beta 8	475253
LAMA3	laminin, alpha 3	480173
LAMB2	laminin, beta 2 (laminin S)	476626
MET	met proto-oncogene (hepatocyte growth factor receptor)	403438
MAPK8	mitogen-activated protein kinase 8	477746
MYLK	myosin light chain kinase	488012
MYLK2	myosin light chain kinase 2	477187
PAK4	p21 protein (Cdc42/Rac)-activated kinase 4	484513
PIK3CA	phosphoinositide-3-kinase, catalytic, alpha polypeptide	488084
PIK3R2	phosphoinositide-3-kinase, regulatory subunit 2 (beta)	609956
PGF	placental growth factor	611916
PDGFD	platelet derived growth factor D	479460
PDGFRB	platelet-derived growth factor receptor, beta polypeptide	442985
PPP1CB	protein phosphatase 1, catalytic subunit, beta isoform	403558
PPP1CC	protein phosphatase 1, catalytic subunit, gamma isoform	403557
PPP1R12A	protein phosphatase 1, regulatory (inhibitor) subunit 12A	475411
RHOA	ras homolog gene family, member A	403954
LOC479252	similar to Vinculin (Metavinculin)	479252
LOC479790	similar to mitogen activated protein kinase 3	479790
TLN1	talin 1	474759
TNC	tenascin C	481689
AKT2	v-akt murine thymoma viral oncogene homolog 2	449021
CRKL	v-crk sarcoma virus CT10 oncogene homolog (avian)-like	608125

**Fig 2:**
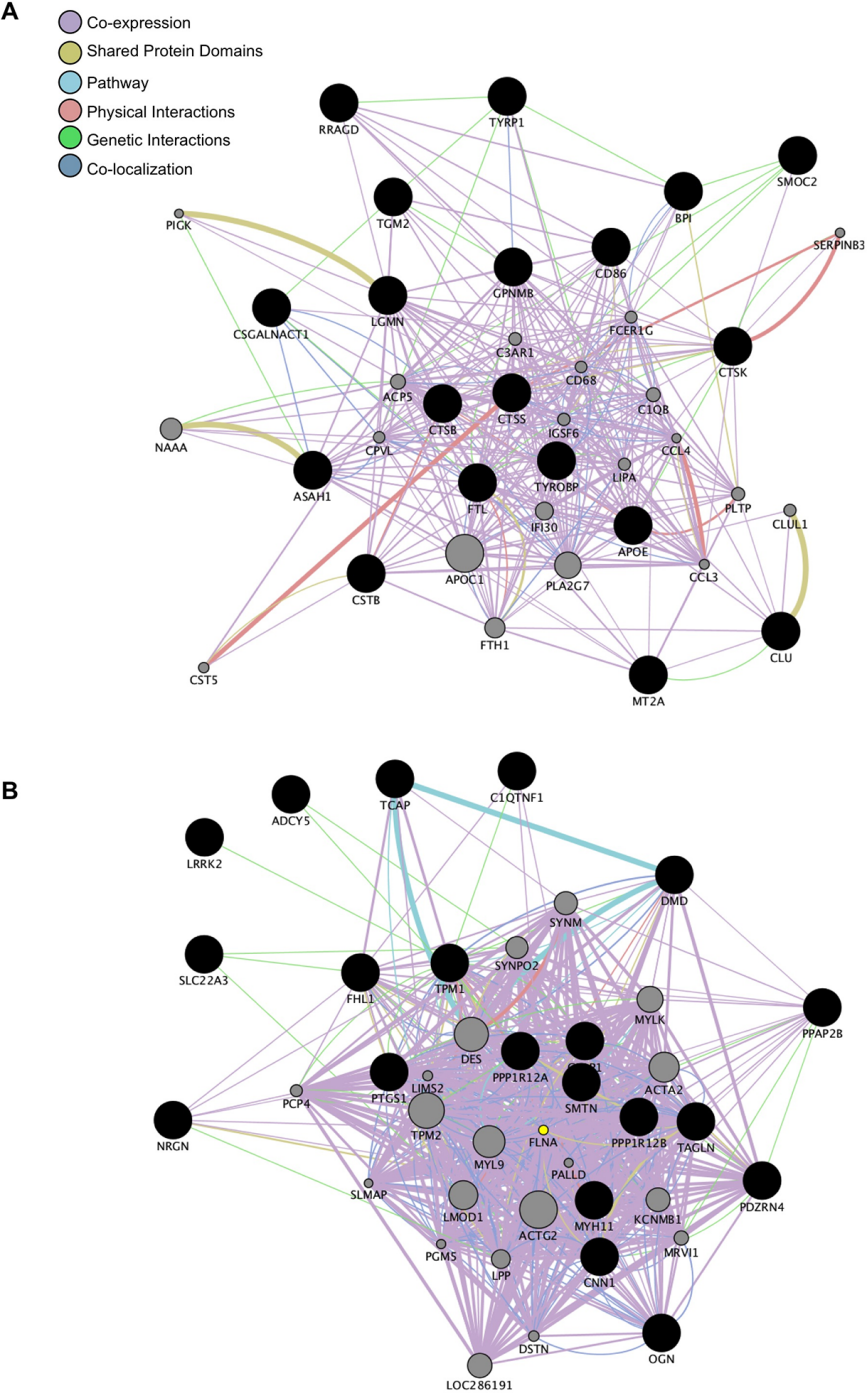
Up- and Downregulated Gene Networks in IDUA^-/-^ Canine Arteries. Gene networks formed using GeneMania using the top related genes and at most 20 attributes. Interactions observed include co-expression, co-localization, genetic interactions, pathway, and physical interactions. A. Network of the upregulated genes. Notable genes in the upregulated network include lysosomal proteases (CTSB, CTSK, CTSS, LGMN); RRAGD, a GTPase involved in lysosomal energy sensing; immunity-related genes such as CD86 and BPI; and clusterin, a protein associated with atherosclerotic cardiovascular disease. B. Network of the downregulated genes. The notable genes in the downregulated network are primarily smooth muscle cytoskeletal elements (CNN1, DMD, MYH11, SMTH, TAGLN, TCAP, and TPN1).

Next, gene networks were constructed, utilizing two methods to identify any potential interactions between genes with perturbed expression. The first was *de novo* using WGCNA, and the second utilized GeneMania. Signed networks were constructed in WGCNA using an adjacency function that incorporates gene correlation and connectivity across the dataset. GeneMania networks were constructed based on known interactions such as co-expression, shared protein domains, signaling pathways, physical interactions, genetic interactions, and co-localization. The results of the GeneMania-generated network analysis are shown in Fig 2, with correlated upregulated genes in the MPS I dogs in Fig 2A: notable genes exceeding 2-fold expression change in the upregulated network include lysosomal proteases (CTSB, CTSK, CTSS, LGMN); RRAGD, a GTPase involved with lysosomal energy sensing; immunity-related genes such as CD86, a macrophage-related signaling protein; BPI, a protein that binds bacterial lipopolysaccharide; and CLU, a marker previously identified in inflammatory processes and atherosclerosis. Fig 2B shows the correlated downregulated (reduced 2-fold) gene network in the MPS I dogs. Many of the downregulated genes are smooth muscle cytoskeletal elements, including TCAP (titin-cap), TPN1 (tropomyosin), DMD (dystrophin), SMTN (smoothelin), TAGLN (transgelin), MYH11 (smooth muscle myosin heavy chain 11), and CNN1 (smooth muscle calponin 1).

**Fig 3:**
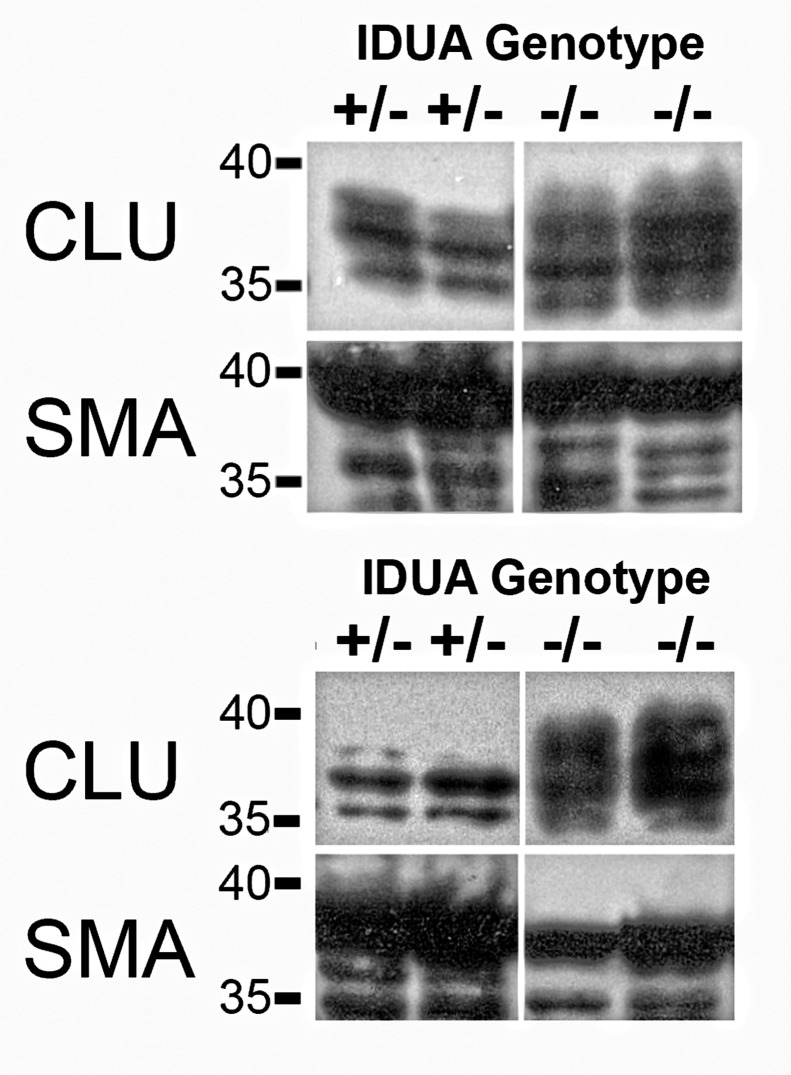
Western Blot of Clusterin and Smooth Muscle Actin in Canine Aortas. Clusterin is predicted to appear as a thicker band between 35–39 kDa owing to differential glycosylation. Alpha smooth muscle actin is predicted to appear at approximately 42 kDa. 20 μg of total protein was loaded in each lane. The upper set is from ascending aorta and the lower set is from descending aorta. Normalized mean clusterin band intensities from descending and ascending aortas are 1.62 and 1.3 fold greater in MPS I animals compared to unaffected animals.

**Fig 4:**
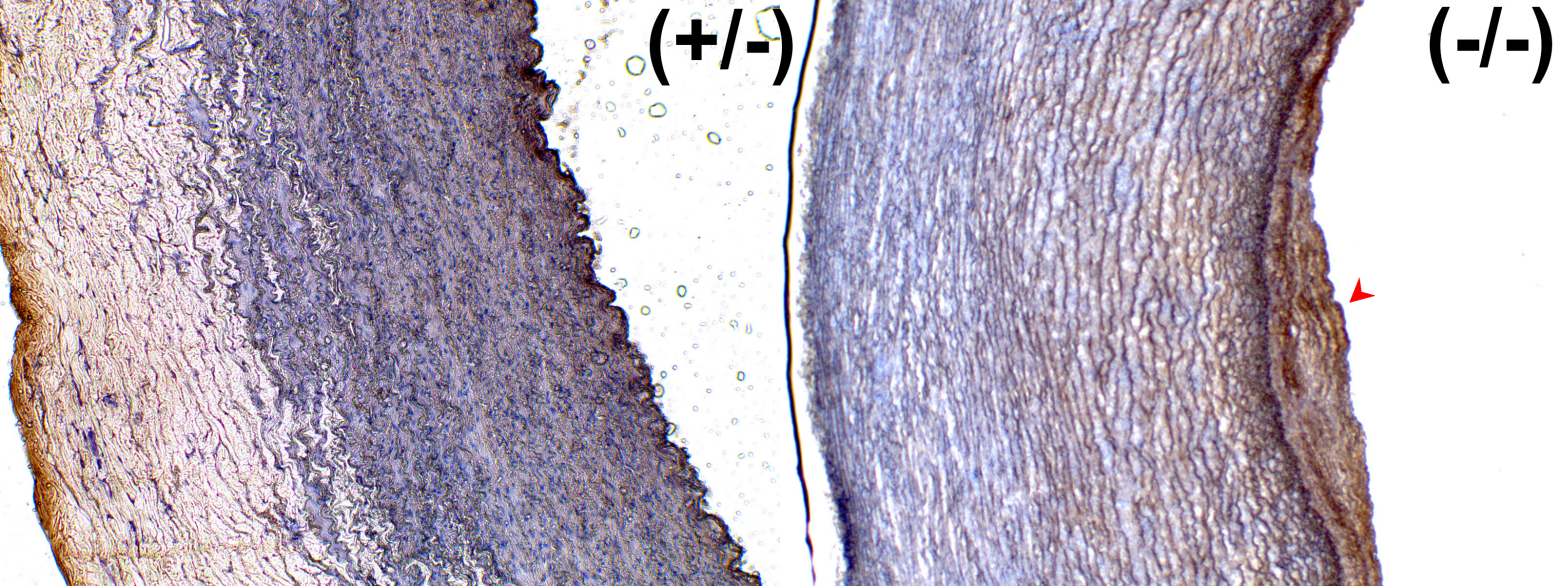
Canine Arterial Clusterin Immunohistochemistry. The MPS I aorta (right panel) has an overall increase in clusterin signal compared to unaffected aorta (left panel), and also contains an eccentric plaque (arrowhead) that stains strongly for clusterin.

We further examined the expression pattern of clusterin, which was present in the upregulated gene network and the most significantly overexpressed gene (S3 Fig). The clusterin overexpression especially interested us, as it has been shown to be is involved in morphologic transformation of vascular smooth muscle cells [48] and present in human atherosclerotic plaques [49]. The MPS I dog aortas contained multiple eccentric plaques that intruded into the lumen, consistent with previous reports of vascular pathology in the model [23]. Importantly, we confirmed that clusterin protein is overexpressed in MPS I ascending (1.3-fold) and descending aortas (1.62-fold) compared to unaffected heterozygous dogs (Fig 3). The clusterin protein is localized primarily within the plaques of the MPS I aortas; little to no clusterin signal was seen in the control aortas (Fig 4). These results provide protein-level confirmation of clusterin gene overexpression within MPS I arterial vasculature. Additionally, Toll-like receptor 4 (TLR4) protein overexpression and increased phosphorylated STAT1 protein were seen only in canine MPS I arteries (S4 Fig).

**Fig 5:**
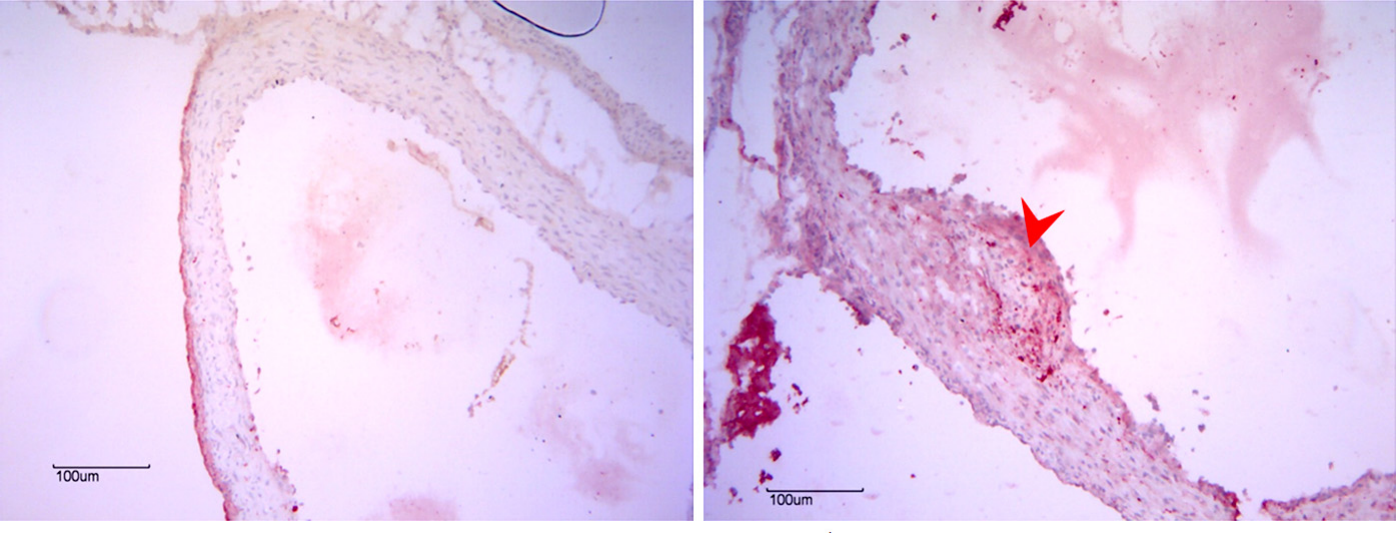
Murine Aortic Clusterin Immunohistochemistry. Taken at the level of the sinuses of Valsalva, the MPS I mouse aorta (right panel) is thicker than the wild-type mouse (left panel) and also contains an eccentric plaque (arrowhead) staining strongly for clusterin.

Next, we assessed whether adult C57/Bl6 *Idua*^-/-^ mice had similar cardiovascular findings to the IDUA^-/-^ dogs, especially with regards to aortic histopathology. The mice had grossly evident hypertrophic cardiomyopathy compared to *Idua*^*+/-*^ control mice, which is consistent with other assessments of cardiovascular phenotype [23]. While not as prominent as the canine MPS I model, the *Idua*^-/-^ mice also demonstrated eccentric, sclerotic plaques and thickened proximal aortas compared to control mice. These plaques were visible only near the sinuses of Valsalva, a distribution different from the canine plaques, which were present throughout the ascending and descending aorta. Clusterin protein was absent in the control mice (Fig 5A), and prominently visible in the aortic plaques of the *Idua*^-/-^ mice (Fig 5B). The full clusterin and smooth muscle actin blots can be viewed in S5 Fig.

## Discussion

We performed microarray analyses comparing arterial gene expression of untreated MPS I canines to unaffected heterozygous canines to gain insights into the gene perturbations caused by α-L-iduronidase deficiency and subsequent arterial glycosaminoglycan storage. Our study represents a comprehensive assessment of gene expression in the vasculature of any MPS model system, and identified upregulation of genes related to lysosomes, proteasomes, macrophages, and innate immunity. We also identified downregulation of cytoskeletal genes and calcium channel subunits. Our findings are consistent with results of gene expression studies from non-vascular tissues in differing MPS subtypes. This indicates the possibility that common mechanisms may be responsible for pathogenesis of MPS disease in multiple organ systems. We also identified evidence linking the innate immune system to pathogenesis of MPS I cardiovascular disease, as well as a biomarker for potentially following disease progression and treatment efficacy.

Overexpression of lysosomal hydrolases and housekeeping genes is consistent with the hypothesis that accumulation of primary substrate in lysosomal storage disorders results in a “traffic jam” with secondarily impaired degradation of other lysosomal substrates, and subsequent efforts by the cell to alleviate the secondary storage [50]. Our findings of cathepsin protease overexpression are consistent with aortic cathepsin B, D, L, and S overexpression from the mouse and canine models of MPS I and VII [31, 33, 34]. In addition, overexpression of lysosomal membrane proteins and enzymes has been observed in murine MPS VII liver [47] and brain [29]. An additional effect of the lysosomal traffic jam is impairment in organelle and protein degradation via a lysosome-dependent pathway known as autophagy [51]. Since the ubiquitin-proteasome system (UPS) is another prominent mechanism for recycling of accumulated proteins, the observed upregulation of proteasomal genes may indicate diversion of protein catabolism towards the UPS.

Downregulation of cellular adhesion and cytoskeletal genes (labeled as “Dilated Cardiomyopathy,” “Focal Adhesion,” and “Arrhythmogenic Right Ventricular Cardiomyopathy" in our study) has also been seen in MPS IIIb mouse brains [30]. These results support data suggesting that excess HS oligosaccharides impair function of cellular adhesion molecules and disrupt normal polarization and orientation of cultured MPSIIIB mouse astrocytes or neural stem cells [52].

The generalized upregulation of macrophage and immunity-related genes observed in the MPS I arteries indicate that inflammation may play a prominent role in the pathogenesis of MPS I cardiovascular disease. This immune-associated gene overexpression was also seen in several expression studies in MPS IIIb mice brains [26, 30] and is consistent with neuroinflammation observed in murine MPS IIIb and other neuropathic lysosomal storage disorders, including Sandhoff Disease and MPS I [53, 54]. While exploring potential etiologies of MPS I cardiovascular disease, significant overexpression of the Toll-like receptor 4 gene, in addition to numerous cathepsin proteases and matrix metalloproteinases was observed [34]. When MPS VII / cathepsin S and MPS VII / MMP-12 double knockout mice continued to develop arterial elastin degradation and aortic root dilatation, it was concluded that other proteases or inflammatory signals transduced by TLR4 activation could be responsible for the MPS VII cardiovascular phenotype [31].

TLR4 is a transmembrane protein that belongs to a family of proteins known as Pattern Recognition Receptors (PRRs). PRRs are expressed by cells of the innate immune system, bind foreign molecules that are typically associated with infections (pathogen-associated molecular patterns or PAMPs) or molecules released by damaged cells (damage-associated molecular patterns or DAMPs), and activate an immune response [55]. Lipopolysaccharide, the canonical PAMP for bacteria, activates TLR4 signaling. Acting via the MyD88 adaptor and STAT1 protein phosphorylation, activated TLR4 results in nuclear translocation of the transcription factor NF-κB and subsequent mRNA expression of proinflammatory cytokines.

Following the discovery that heparan sulfate, but neither chondroitin sulfate nor heparin, activates TLR4 signaling in dendritic cells and macrophages, evidence has been accumulating that heparan sulfate may play a role in the pathogenesis of MPS neurologic and orthopedic disease via TLR4-induced inflammation [56–58]. Wild-type mouse microglia respond in a dose-dependent fashion to HS-oligosaccharides isolated from MPS IIIB patients with overexpression of macrophage inflammatory protein 1α (*Mip1a* or *Ccl3*) and interleukin 1β mRNAs. There was significantly reduced cytokine mRNA expression when the same HS-oligosaccharides were administered to *Tlr4*^-/-^ or *MyD88*^-/-^ mouse microglia. MPS IIIb (*Naglu*^-/-^) mouse brains overexpress *Mip1a* mRNA as early as postnatal day 10 with concomitant microglial activation and neuroinflammation. *Naglu* / *Tlr4* and *Naglu* / *MyD88* double knockout mice did not overexpress *Mip1a* and demonstrated delayed neuroinflammation [33]. Similar neuroinflammatory findings have been reported in MPS IIIa, IIIb, and IIIc mice, all of which accumulate HS [28].

Our results suggest that HS–TLR4 mediated, monocyte/macrophage-induced inflammation contributes to the pathogenesis of cardiovascular disease in MPS I. Among the genes with highest overexpression in MPS I arteries was CD86 which, in conjunction with another overexpressed gene CD80, encode proteins found on the cell surface of activated monocytes. Other immunity-related genes with observed overexpression were members of the major histocompatibility complex II families, which are involved in antigen presentation and are found primarily on monocytes, macrophages, and dendritic cells. In addition, overexpression of the inflammatory cytokines interleukin-1α, interleukin-1β, interleukin 2, and interleukin 6 were observed. We also identified MPS I arterial overexpression of TLR4 protein and evidence of its activation via increased levels of phosphorylated STAT1.

Heparan sulfate-mediated inflammation cannot be the sole etiology of MPS cardiovascular disease pathogenesis. Patients who have MPS type VI store only DS GAG but often develop mitral or aortic valve dysfunction severe enough to necessitate surgical valve replacement, and also demonstrate coronary artery intimal thickening with arterial stiffness [2, 19,59]. Although the effects of DS GAG on TLR4 in MPS have not been directly investigated, there are studies that indicate it may also serve as a PAMP for TLR4 and promote inflammation like HS GAGs [60, 61]. Post-mortem studies of several MPS IVa patients, who store neither HS nor DS but accumulate keratan and chondroitin sulfate GAGs, have also demonstrated coronary sclerosis or aortic intimal thickening with macrophage infiltration and elastin fibril disruption [62, 63].

As our primary focus was canine MPS I arterial disease, mRNA and protein quantification of murine aorta clusterin or pSTAT1 were not performed. Despite this limitation, our study provides evidence from both canine and murine models confirming clusterin protein overexpression within MPS I arterial lesions. We believe that clusterin may have utility as an *in vivo* biomarker of MPS I cardiovascular disease for several reasons. First, overexpression of clusterin has been observed in many inflammatory conditions, including human atheromatous atherosclerosis, where it is observed only in vascular smooth muscle cells and stroma of atheromas and not in normal aorta [64, 65]. Second, while the effects of clusterin overexpression on human cardiovascular disease are unclear and may be pro-atherosclerotic [66] or protective [67, 68], clusterin serum levels correlate with severity of coronary artery stenosis and are highest during active myocardial infarction [69, 70]. Although lipid-mediated atheroma formation and atherosclerosis appear to proceed via pathways distinct from what occurs in MPS I, the roles of macrophages, inflammation, and Toll-like receptors in promoting atherosclerosis are well known [71]. Further studies are required to determine if circulating clusterin levels are indeed elevated in human MPS I patients, and if those levels correlate with severity of their cardiovascular disease.

Because GAGs are naturally present in healthy aortas [72], it will be important in the design of MPS cardiovascular disease therapeutics to determine if the arterial inflammation is caused solely by a quantitative increase of GAGs, or if the GAGs stored in MPS possess additional qualitative pro-inflammatory effects. The presence of storage-induced inflammation within MPS I canine arteries suggests several possible therapeutic strategies. Modified IDUA enzymes are being developed that have the potential to permeate deeper into the vascular parenchyma to alleviate GAG storage. An alternative strategy is the development of small molecule therapies that diffuse into arterial parenchyma and block the inflammatory response leading to MPS cardiovascular disease via inhibition of TLR4 signaling or pro-inflammatory cytokines. In fact, preclinical studies have been performed to investigate each of these modalities [73–76], and human clinical trials are in progress (European Union Clinical Trials Register # 2014-000350-11). Clusterin represents a promising biomarker to monitor efficacy of these therapeutic candidates in both preclinical and clinical studies for MPS I cardiovascular disease. The murine MPS I model system can be utilized to test efficacy of potential therapeutics targeted towards MPS cardiovascular disease.

## Supporting Information

S1 Appendix Protocol for murine *Idua* genotyping. This protocol was utilized for identifying the *Idua* genotype of the mice utilized in this study.
(PDF)

S1 Fig Dendrogram and trait heatmap of the IDUA^-/-^ and control canine aorta samples. The control samples cluster together and the IDUA^-/-^ canine aorta samples cluster together. The same results are found if data from each tissue type is analyzed separately for up- and down-regulated genes, respectively.
(TIF)

S2 Fig Module trait-relationship table. Modules are designated on the left and the trait relationship is on the bottom (A for ascending aorta, C for carotid artery, and D for descending aorta). The table shows the correlation (top value) and the significance (bottom value) for each module-trait relationship. The turquoise and blue modules show the highest significance and thus were used for further analysis.
(TIF)

S3 Fig Identity of top perturbed genes within modules. In the turquoise and blue modules, further assessment identified the top genes based on fold- change and p-value using Student’s t-test. A. Volcano plot of the–log_10_ of the p-value vs. log_2_ of fold change. B. Top up- and down-regulated genes in the module are represented in a bar graph with a log_2_ scale.
(TIF)

S4 Fig TLR4 and pSTAT1 immunohistochemistry. Immunohistochemistry for demonstrates overexpression of TLR4 in canine MPS I aorta, but not in unaffected canine aorta. The presence of increased pSTAT1 in canine MPS I aorta compared to unaffected canine aorta is evidence for TLR4 activation.
(TIF)

S5 Fig Clusterin Western blotting. Scan of raw radiograph (Thermo Scientific CL-XPosure Film, ThermoFisher Scientific, Waltham, MA) exposure depicting enhanced chemiluminescence detection of canine aorta WB probed with anti-human clusterin alpha chain (EMD Millipore, Billerica, MA) and with anti-human smooth muscle alpha-actin (Dako North America, Inc., Carpinteria, CA). Hand written annotations refer to specific animal identifiers marking the lanes. Samples from descending or ascending aorta are grouped together and labeled as IDUA^+/-^ (carrier) or IDUA^-/-^ (MPS I). Handwritten markings indicate size and position of color coded protein ladder size markers (PageRuler Prestained Protein Ladder, Life Technologies, Grand Island, NY) traced by overlaying the film onto the transfer membrane. Clusterin is predicted to appear as a band between 35–39 kDa owing to differential glycosylation, while alpha smooth muscle actin is predicted to appear at approximately 42 kDa.
(TIF)

S1 Table Significantly upregulated genes within IDUA^-/-^ canine aorta. The full list of mRNA probes showing significant (p-value ≤ 0.05) and greater than 2-fold level of upregulation in IDUA^-/-^ canine arteries, compared to controls. Note that some genes are represented more than once due to existence of multiple mRNA probes per gene.
(XLSX)

S2 Table Significantly down-regulated genes within IDUA^-/-^ canine aorta. The full list of mRNA probes showing significant (p-value ≤ 0.05) and greater than 2-fold level of down-regulation in IDUA^-/-^ canine arteries, compared to controls. Again, some genes are represented more than once due to existence of multiple mRNA probes per gene.
(XLSX)
