# Supplementary material for: Immune-Mediated Inflammation May Contribute to the Pathogenesis of Cardiovascular Disease in Mucopolysaccharidosis Type I
Source: PLoS One. 2016 Mar 17;11(3):e0150850. doi: 10.1371/journal.pone.0150850 (PMC4795702; doi:10.1371/journal.pone.0150850)
Supplement: S1 Appendix — This protocol was utilized for identifying the Idua genotype of the mice utilized in this study. (PDF) [file pone.0150850.s001.pdf]

MPSI Genotyping Protocol

Primer 1: GGAACTTTGAGACTTGGAATGAACCAG
Primer 2: CATTGTAAATAGGGGTATCCTTGAACTC
Primer 3: GGATTGGGAAGACAATAGCAGGCATGCT

PCR

	1X
H_2O	10.9 µL
Buffer Q	4 µL
10X Buffer	2 µL
$MgCl_2$	0.5 µL
Primer 1 (10uM)	2 µL
Primer 2 (10uM)	2 µL
Primer 3 (10uM)	2 µL
DNTP	0.5 µL
Taq Polymerase	0.1 µL

Add 24ul / tube

PCR Reaction

First Denaturing	95°C	15min
Denature<br>Anneal<br>Extention	95°C<br>57°C<br>72°C	55sec<br>44sec<br>1min 30 sec
Hold	10°C	
(repeat boxed set 35X)		

WT band = 500bp
Knock out band = 350 bp
