# Supplementary figures and images for: Immune-Mediated Inflammation May Contribute to the Pathogenesis of Cardiovascular Disease in Mucopolysaccharidosis Type I
Source: PLoS One. 2016 Mar 17;11(3):e0150850. doi: 10.1371/journal.pone.0150850 (PMC4795702; doi:10.1371/journal.pone.0150850)

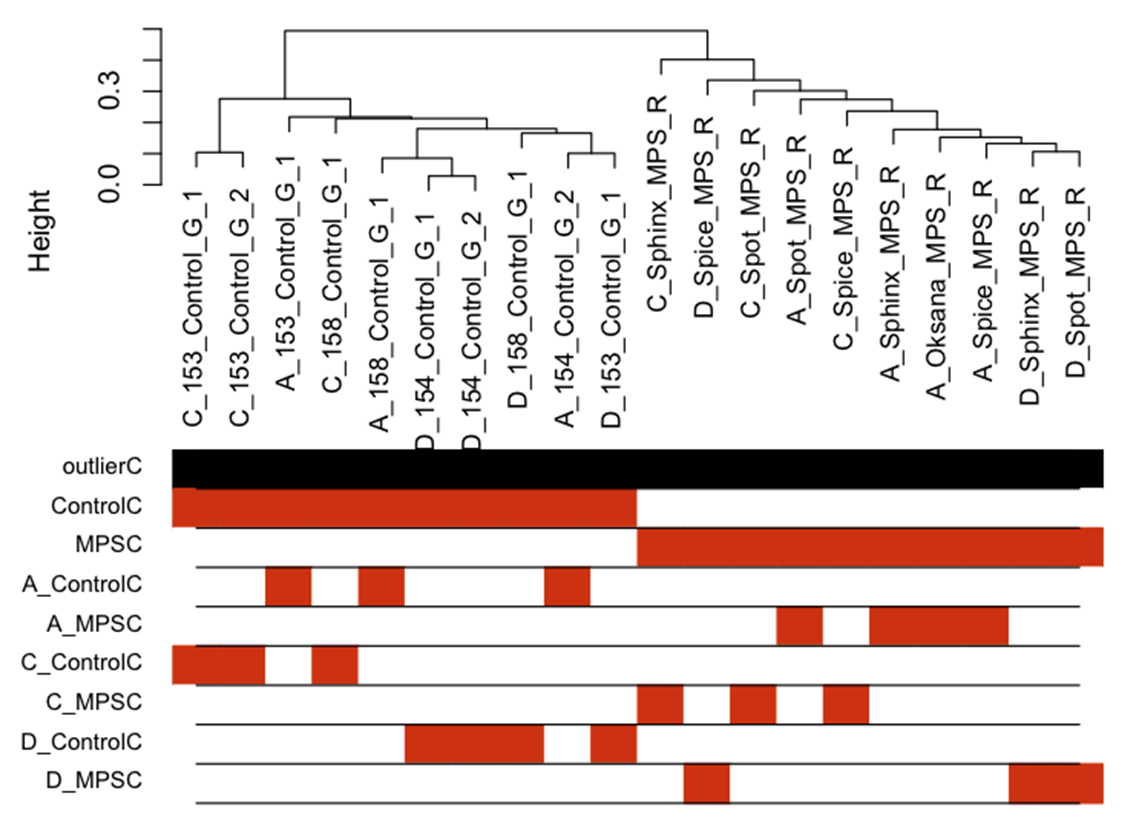

Supplement: S1 Fig — The control samples cluster together and the IDUA-/- canine aorta samples cluster together. The same results are found if data from each tissue type is analyzed separately for up- and down-regulated genes, respectively. (TIF) [file pone.0150850.s002.tif]

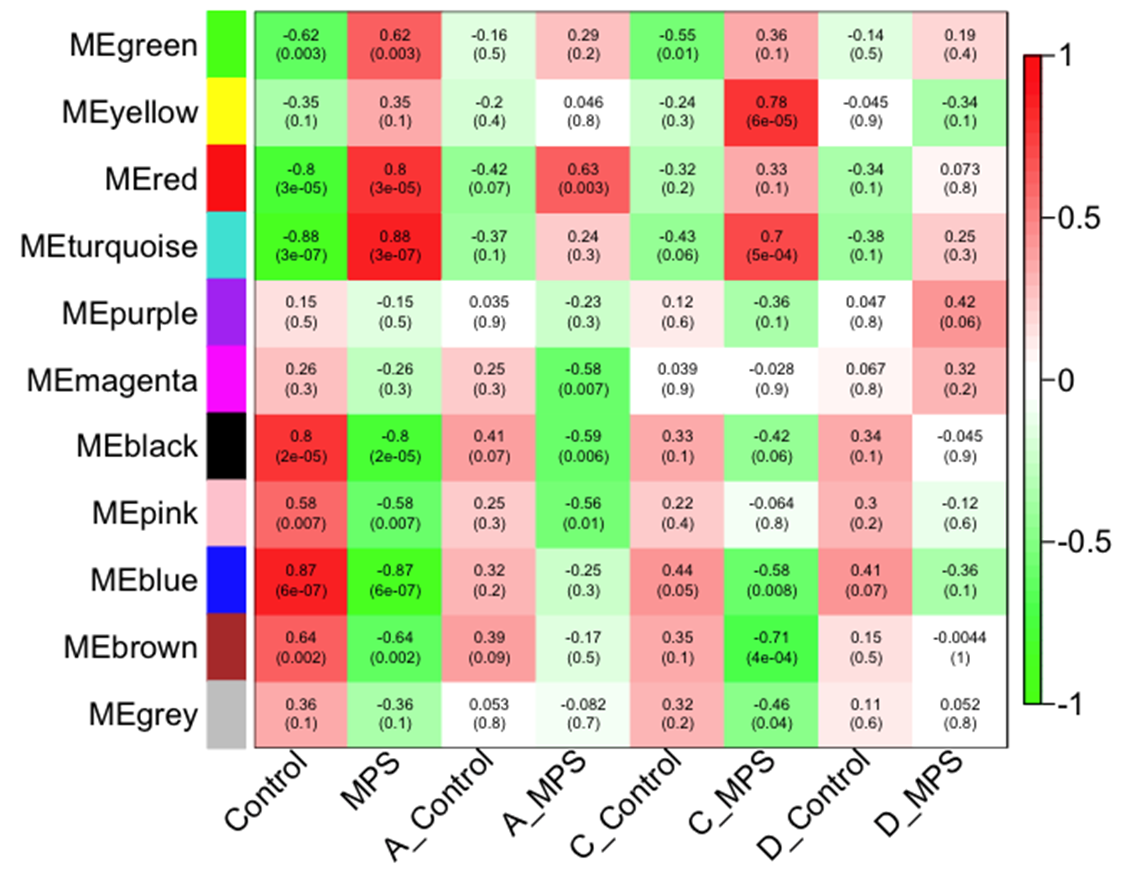

Supplement: S2 Fig — Modules are designated on the left and the trait relationship is on the bottom (A for ascending aorta, C for carotid artery, and D for descending aorta). The table shows the correlation (top value) and the significance (bottom value) for each module-trait relationship. The turquoise and blue modules show the highest significance and thus were used for further analysis. (TIF) [file pone.0150850.s003.tif]

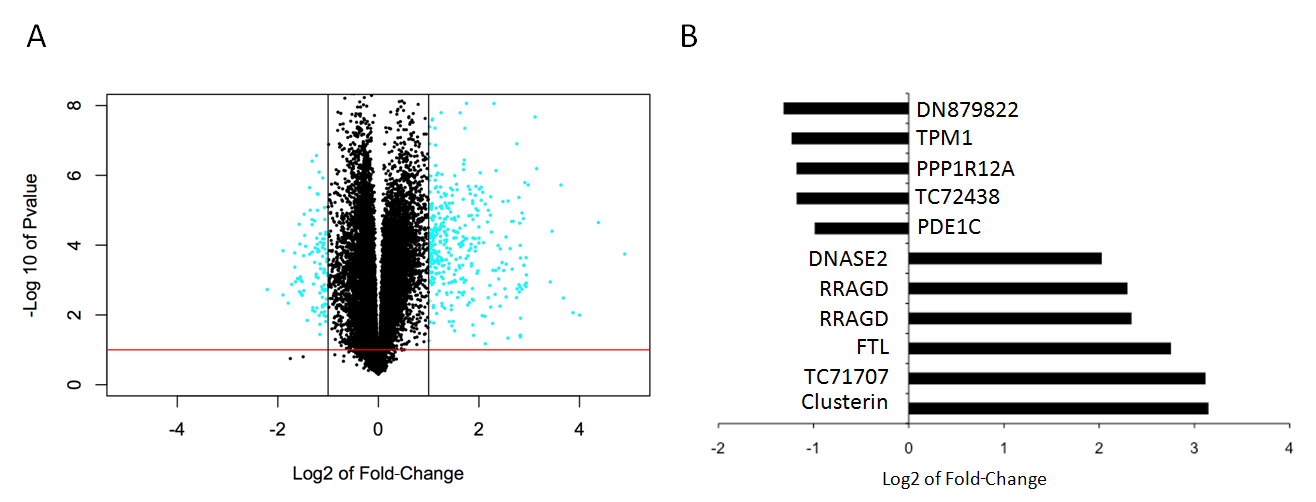

Supplement: S3 Fig — In the turquoise and blue modules, further assessment identified the top genes based on fold- change and p-value using Student’s t-test. A. Volcano plot of the–log10 of the p-value vs. log2 of fold change. B. Top up- and down-regulated genes in the module are represented in a bar graph with a log2 scale. (TIF) [file pone.0150850.s004.tif]

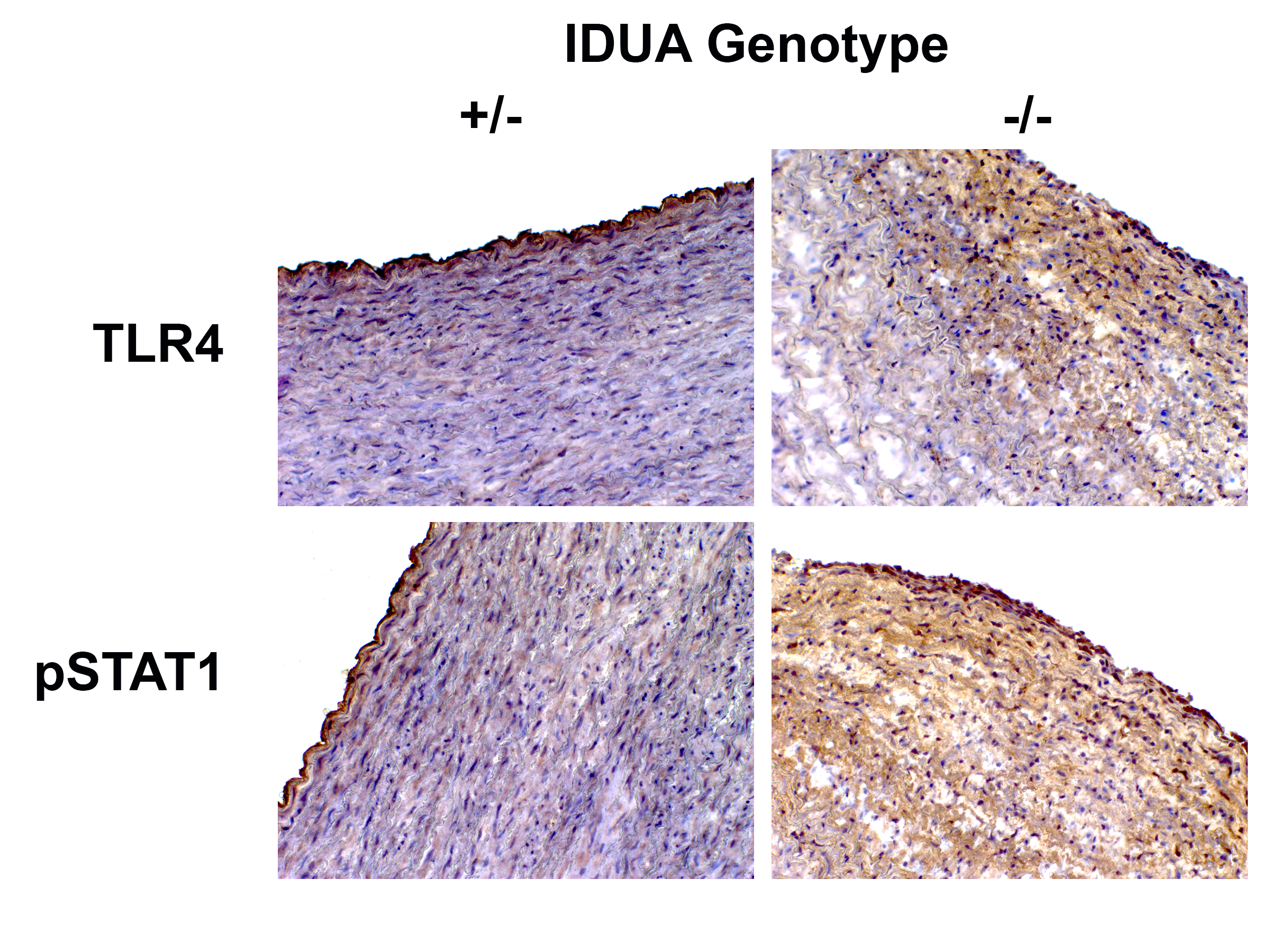

Supplement: S4 Fig — Immunohistochemistry for demonstrates overexpression of TLR4 in canine MPS I aorta, but not in unaffected canine aorta. The presence of increased pSTAT1 in canine MPS I aorta compared to unaffected canine aorta is evidence for TLR4 activation. (TIF) [file pone.0150850.s005.tif]

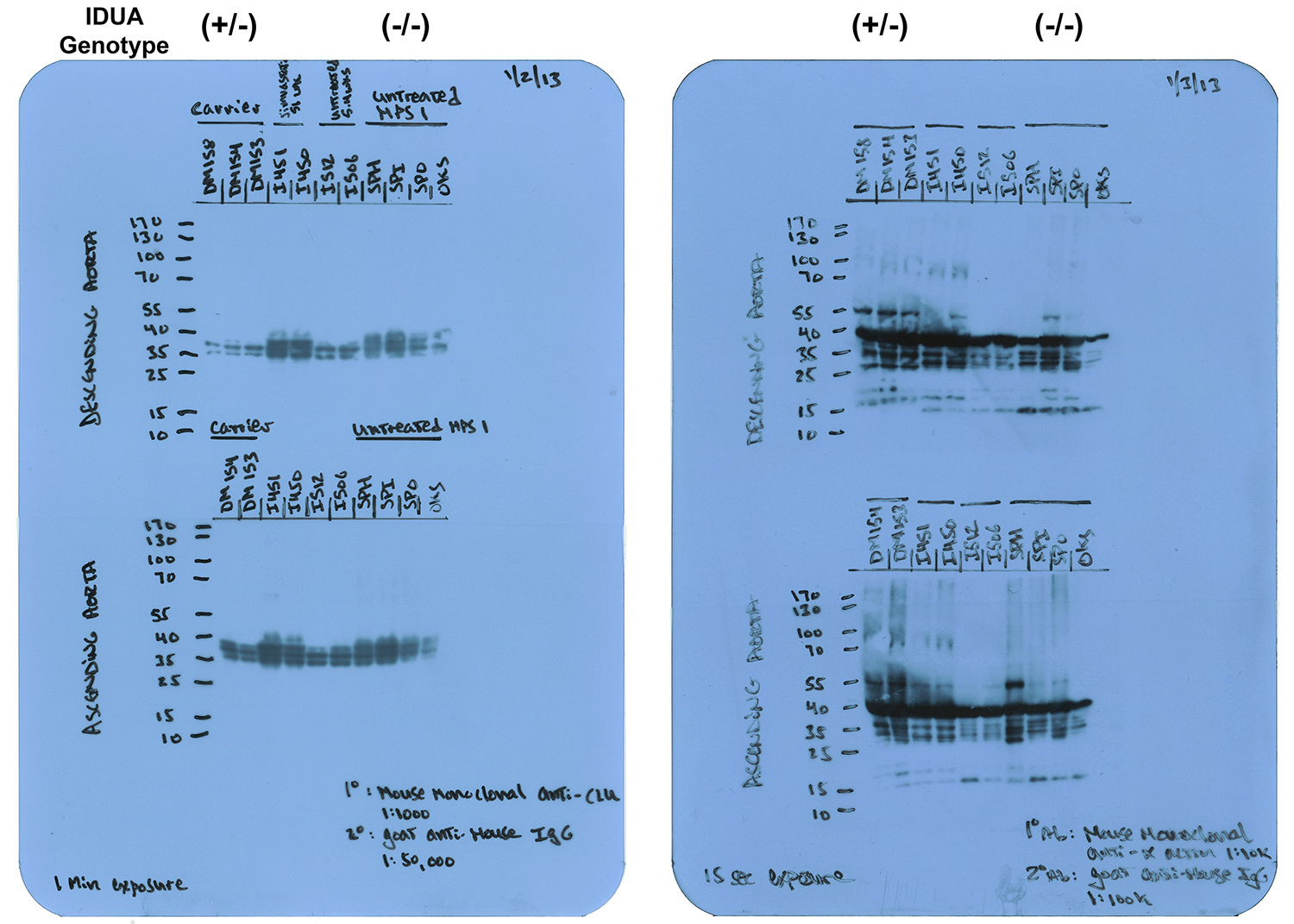

Supplement: S5 Fig — Scan of raw radiograph (Thermo Scientific CL-XPosure Film, ThermoFisher Scientific, Waltham, MA) exposure depicting enhanced chemiluminescence detection of canine aorta WB probed with anti-human clusterin alpha chain (EMD Millipore, Billerica, MA) and with anti-human smooth muscle alpha-actin (Dako North America, Inc., Carpinteria, CA). Hand written annotations refer to specific animal identifiers marking the lanes. Samples from descending or ascending aorta are grouped together and labeled as IDUA+/- (carrier) or IDUA-/- (MPS I). Handwritten markings indicate size and position of color coded protein ladder size markers (PageRuler Prestained Protein Ladder, Life Technologies, Grand Island, NY) traced by overlaying the film onto the transfer membrane. Clusterin is predicted to appear as a band between 35–39 kDa owing to differential glycosylation, while alpha smooth muscle actin is predicted to appear at approximately 42 kDa. (TIF) [file pone.0150850.s006.tif]
